# BCATc inhibitor 2 ameliorated mitochondrial dysfunction and apoptosis in oleic acid-induced non-alcoholic fatty liver disease model

**DOI:** 10.3389/fphar.2022.1025551

**Published:** 2022-10-28

**Authors:** Zhuo Lu, Gui-Feng Sun, Xiao-An Pan, Xin-Hui Qu, Ping Yang, Zhi-Ping Chen, Xiao-Jian Han, Tao Wang

**Affiliations:** ^1^ Institute of Geriatrics, Jiangxi Provincial People’s Hospital and The First Affiliated Hospital of Nanchang Medical College, Nanchang, China; ^2^ Department of Thoracic Surgery, The First Affiliated Hospital of Nanchang University, Nanchang, China; ^3^ Department of Pharmacology, School of Pharmaceutical Science, Nanchang University, Nanchang, China; ^4^ Department of Neurology, Jiangxi Provincial People’s Hospital and The First Affiliated Hospital of Nanchang Medical College, Nanchang, China; ^5^ Department of Critical Care Medicine, Jiangxi Provincial People’s Hospital and The First Affiliated Hospital of Nanchang Medical College, Nanchang, China

**Keywords:** NAFLD, BCTAc inhibitor 2, mitochondrial dysfunction, apoptosis, oxidative stress

## Abstract

Nonalcoholic fatty liver disease (NAFLD) is a prevalent hepatic disease in the world. Disorders of branched chain amino acid (BCAA) metabolism is involved in various diseases. In this study, we aim to explore the role of BCAA metabolism in the development of NAFLD and the protective effect of BCATc Inhibitor 2, an inhibitor of cytosolic branched chain amino acid transaminase, against NAFLD as well as its underlying mechanism. It was found that oleic acid induced lipid accumulation and apoptosis in HepG2 and LO2 cells. Supplementation of BCAAs further aggravated oleic acid-induced lipid accumulation and apoptosis. In contrast, treatment of BCATc Inhibitor 2 ameliorated oleic acid-induced lipid accumulation and apoptosis. Molecularly, supplementation of BCAAs or treatment of BCATc Inhibitor 2 up-regulated or down-regulated the expression of SREBP1 and lipogenesis-related genes without affecting lipolysis-related genes. BCATc Inhibitor 2 maintained mitochondrial function by ameliorating oleic acid-induced mitochondrial ROS generation and mitochondrial membrane potential disruption. In addition, BCATc Inhibitor 2 treatment alleviated oleic acid-induced activation of JNK and AKT signaling pathway and Bcl2/Bax/Caspase axis. In conclusion, our results indicate BCAA metabolism is involved in NAFLD and BCATc Inhibitor 2 protects against oleic acid-induced lipid accumulation and apoptosis. These findings suggest that BCATc Inhibitor 2 is a promising candidate drug for the treatment of NAFLD.

## Introduction

Due to the rising prevalence of obesity, nonalcoholic fatty liver disease (NAFLD) is now the most common liver disease in the world. The clinicopathological syndrome of NAFLD ranges from simple hepatic steatosis to nonalcoholic steatohepatitis (NASH), and NASH can evolve to liver cirrhosis and hepatocellular carcinoma (Loomba and Sanyal, 2013). The original 2-hit hypothesis suggested that triglyceride accumulation and inflammatory response were the main drives of NAFLD (Boden, 1997). Recently, it has been proposed that multiple pathways are interrelated and contribute to NASH. Insulin resistance is closely associated with NASH, since more than 70% diabetic and obese patients have NASH (Loomba et al., 2012). Obesity resulted from overnutrition is the common cause of NASH. In obese individuals, excessive free fatty acids are uptake by liver, which induces gluconeogenesis and lipogenesis (Boden, 1997). Adipocytokines such as adiponectin and leptin are key to maintain metabolic homeostasis in adipose tissue. Down-regulated level of adiponectin and up-regulated level of leptin were observed in the serum of NASH patients (Poordad, 2004; Masarone et al., 2014). While *de novo* lipogenesis contributes to 5% accumulation of cholesterol (TG) in health individuals, it is significantly elevated in NASH individuals (Basaranoglu et al., 2015). Intervening lipogenesis and its related metabolic pathways proved to a promising strategy in the treatment of NAFLD.

Apoptosis, a process of programmed cell death, is a genetically and organized controlled form of cell death. Elevated apoptosis in hepatocytes is a hallmark of various liver diseases, including viral hepatitis, cholestasis, toxicant-induced injury and ischemia/reperfusion (Wang et al., 2020; Zheng et al., 2020; Ghanbarinejad et al., 2021). Lipotoxicity induced by lipids accumulating could result in apoptotic cell death, which is a key feature of NAFLD (Kusminski et al., 2009). It was reported that compared with alcoholic hepatitis or simple steatosis patients, apoptosis significantly increases in the livers of NASH patients (Kanda et al., 2018). STING/IRF3 signaling could promote hepatocyte inflammation and apoptosis in NAFLD (Qiao et al., 2018). It was showed that activation of Fas/FasL and subsequent Caspase 8 contributed to NASH (Li et al., 2014). Thus, inhibiting hepatic apoptosis by pharmacological drugs has been proposed in NASH therapy (Witek et al., 2009; Li et al., 2020).

/Branched chain amino acids (BCAAs), comprising of valine (Val), isoleucine (Ile) and leucine (Leu), are essential amino acids that take up about 15–25% of total protein intake (Layman, 2003). Aside from protein synthetic, BCAAs could act as critical nutrition signals in the regulation of metabolism and energy homeostasis (Lynch and Adams, 2014). Branched-chain amino acid transferase (BCAT), including cytosolic BCATc and mitochondrial BCATm, catalyzes the transamination of BCAAs, which is the first step in BCAA catabolism. While BCATm expressed ubiquitously, the expression of BCATc is limited in brain, ovary, peripheral nervous system, kidney, and to a lesser extent in liver (Suryawan et al., 1998). Recently, the significant role of BCAT mediated metabolic reprogramming in multiple diseases was indicated in a growing number of studies, including tumor, inflammatory diseases, and T2DM (Nie et al., 2018). Previous study showed that BCATc is overexpressed in patients with NAFLD (Wegermann et al., 2018). Supplementation of dietary BCAAs exacerbated obesity-related lipid metabolism disorders (Zhao et al., 2020). Therefore, BCAAs catabolism and BCAT could be potential therapeutic target for NAFLD.

Coumarin is a polyphenolic compound that was first isolated from *Dipteryx odorata* (Murray et al., 1982). There are approximately 800 coumarin and coumarin derivatives that could be naturally isolated from vascular plants (Cooke et al., 1997). Recent studies showed that coumarin and coumarin derivatives had various biological activity, including anti-inflammatory (Tosun et al., 2009), antibacterial (Musicki et al., 2000) and antitumor effect (Vianna et al., 2012). Aside from medical use, coumarin and coumarin derivatives could be used as prime materials in synthesis of pharmaceuticals. BCATc Inhibitor 2 is an inhibitor of BCATc, which can be synthesized by incorporating 2-CF_3_-phenyls into coumarin derivative (Caballero et al., 2009). It was reported that BCATc Inhibitor 2 demonstrated neuroprotective efficacy *in vivo* and was a potential drug in the treatment of neurodegenerative diseases (Hu et al., 2006).

In this study, we aim to explore the role of BCAAs metabolism in NAFLD and the protective effect of BCATc Inhibitor 2 in oleic acid (OA)-induced formation of lipid droplet and apoptosis in hepatic cells. It was found that supplementation of BCAAs promoted lipid accumulation and apoptosis in oleic acid-induced LO2 and HepG2 cells. Inhibiting BCAAs metabolism by BCATc Inhibitor 2 (INc), an inhibitor of BCATc, alleviated oleic acid-induced formation of lipid droplet and apoptosis. Molecularly, BCATc Inhibitor 2 treatment decreased the expression of SREBP1 and lipogenesis-related genes without affecting lipolysis-related genes. BCATc Inhibitor 2 preserved mitochondrial function by ameliorating oleic acid-induced intracellular ROS generation and mitochondrial membrane potential disruption. In addition, BCATc Inhibitor 2 treatment attenuated oleic acid-induced activation of AKT/ERK signaling and Bcl2/Bax/Caspase axis. In conclusion, our findings indicate that BCATc Inhibitor 2 protects against oleic acid-induced lipogenesis and apoptosis in hepatic cells and demonstrate that BCATc Inhibitor 2 is a promising drug for the treatment of NAFLD in clinic.

## Materials and methods

### Cell culture

Human hepatic cell lines LO2 and HepG2 were purchased from Chinese Academy of Cell Resource Center (Shanghai, China). Cells were cultured in DMEM (Genview, GD3123) supplemented with 10% fetal bovine serum (FBS; Gibco) and maintained at 37°C containing 5% CO_2_.

### Reagents

Oleic acid (#01008) and oil red O (#O0625) were purchased from Sigma. BCATc Inhibitor 2 (#9002002) was purchased from Cayman Chemical. BCAT-IN-2 (#HY-141669) was purchased from MedChemExpress.

### Oil red O staining

LO2 and HepG2 cells were cultured in serum-free medium for 12 h and then cultured in normal medium supplemented with 0.5 mM oleic acid-bovine serum albumin complex for 48 h. Cells were washed with PBS twice and fixed with 4% paraformaldehyde for 1 h. Then, cells were incubated with oil red O solution for 1 h in darkness and photographed with a light microscope.

### Cell viability assay

HepG2 or LO2 cells were seed in 96-well plate and treated with indicated reagents. Then, 10 μL MTT solution was added to each well and incubated at 37°C for 4 h in darkness. Formazan crystals were dissolved with dimethyl sulfoxide and absorbance at 570 nm was detected.

### Measurement of TG and TC levels

Intracellular TG and TC content was determined with Triglycerides Assay Kit (Nanjin Jiancheng Bioengineering Institute, F001-1-1) and Total cholesterol Assay Kit (Nanjin Jiancheng Bioengineering Institute, F002-1-1) according to manufacturer’s instructions.

### RNA extraction and qPCR analysis

Total RNA of hepatic cell lines was extracted with TRIzol reagent (15596-026, Invitrogen) and reverse transcribed to cDNA using Prime RT reagent kit (RR047A, Takara). qPCR analysis was performed with SYBR Green Premix Ex Taq II kit (Takara, RR820A) and ABI ViiA^TM^ 7 Real-Time PCR System. The sequences of primers used were as followings: ACLY 5′-GAC​TTC​GGC​AGA​GAC​AGG​TAG-3′ (sense) and 5′-TCA​GGA​GTG​ACC​CGA​GCA​TA-3′ (antisense), FASN 5′-GGA​TCA​CAG​GGA​CAA​CCT​GG-3′ (sense) and 5′-GCT​GTG​GTC​CCA​CTT​GAT​GA-3′ (antisense), ACC 5′-GCC​TCT​CAG​CTG​GTC​AGA​TTC-3′ (sense), and 5′-CTG​GTT​CAG​CTC​CAG​AGG​TT-3′ (antisense), SCD1 5′-AGC​AGG​TAA​ATT​GTC​GGG​GG-3′ (sense), and 5′-ACT​TTT​TAC​CCC​GAG​CCA​GG-3′ (antisense), ATGL 5′-TGA​GAG​GGG​AGG​TTT​CCA​CA-3′ (sense), and 5′-CAG​CAG​GCC​ATG​AAA​AAC​GG-3′ (antisense), HSL 5′-AAC​CCA​AGA​GGA​AGT​GCC​AT-3′ (sense), and 5′-GCT​CTA​GCG​GGG​TTA​TAG​GC-3′ (antisense).

### Cell apoptosis assay

Cell apoptosis assay was determined with Annexin V-FITC/PI apoptosis kit (BD Biosciences, 556547). In summary, cells were washed with PBS twice and resuspended in 300 μL binding buffer. Subsequently, cells were incubated with Annexin V-FITC and PI for 20 min in the dark. Apoptotic cell rate was then analyzed with flow cytometry (FACS Canto® II, BD).

### Detection of mitochondrial ROS level

After indicated treatment, cells were washed with PBS twice and incubated with MitoSOX (Invitrogen) at final concentration of 5 μM in the dark for 10 min at 37°C. Cells were then washed with PBS and photograph was obtained with a converted fluorescence microscope.

### Detection of mitochondrial membrane potential

After indicated treatment, cells were washed with PBS twice and incubated with JC-1 dye (Thermo Fisher) at the final concentration of 2 μM at 37°C for 15 min. The fluorescence was detected under fluorescence microscope (Olympus, Tokyo, Japan).

### Western blot

Proteins were separated on 10% or 12% SDS-PAGE gel and transferred to PVDF membranes (Millipore, IPVH00010), which were blocked with 5% BSA (Genview, FA016). Next PVDF membranes were incubated with the following antibodies: SREBP1 (PA1-337, ThermoFisher), Bax (50599-2-AP, Proteintech), Bcl2 (12789-1-AP, Proteintech), Caspase 3 (19677-1-AP, Proteintech), Caspase 9 (#9502, Cell Signaling Technology), AKT (#4691, Cell Signaling Technology), p-AKT (#4060, Cell Signaling Technology), JNK (10023-1-AP, Proteintech), p-JNK (80024-1-RR, Proteintech) and β-Actin (AC004, Abconal). After washed 3 times with TBST buffer, PVDF membranes were then incubated with horseradish peroxidase-conjugated anti-rabbit or anti-mouse secondary antibodies (Thermo Fisher Scientific). Western blot band were visualized by digital gel image analysis system (TANON 5500) and Pro-Light chemiluminescence detection kit (TIANGEN, PA112-01).

### Statistical analysis

All the data were showed as mean ± SD. Each experiment were performed at least three times. Differences between groups were calculated by Student’s *t* test or ANOVA where appropriate. *P* < 0.05 were considered significant.

## Results

### BCAA metabolism contributes to OA-induced hepatic steatosis

**FIGURE 1:**
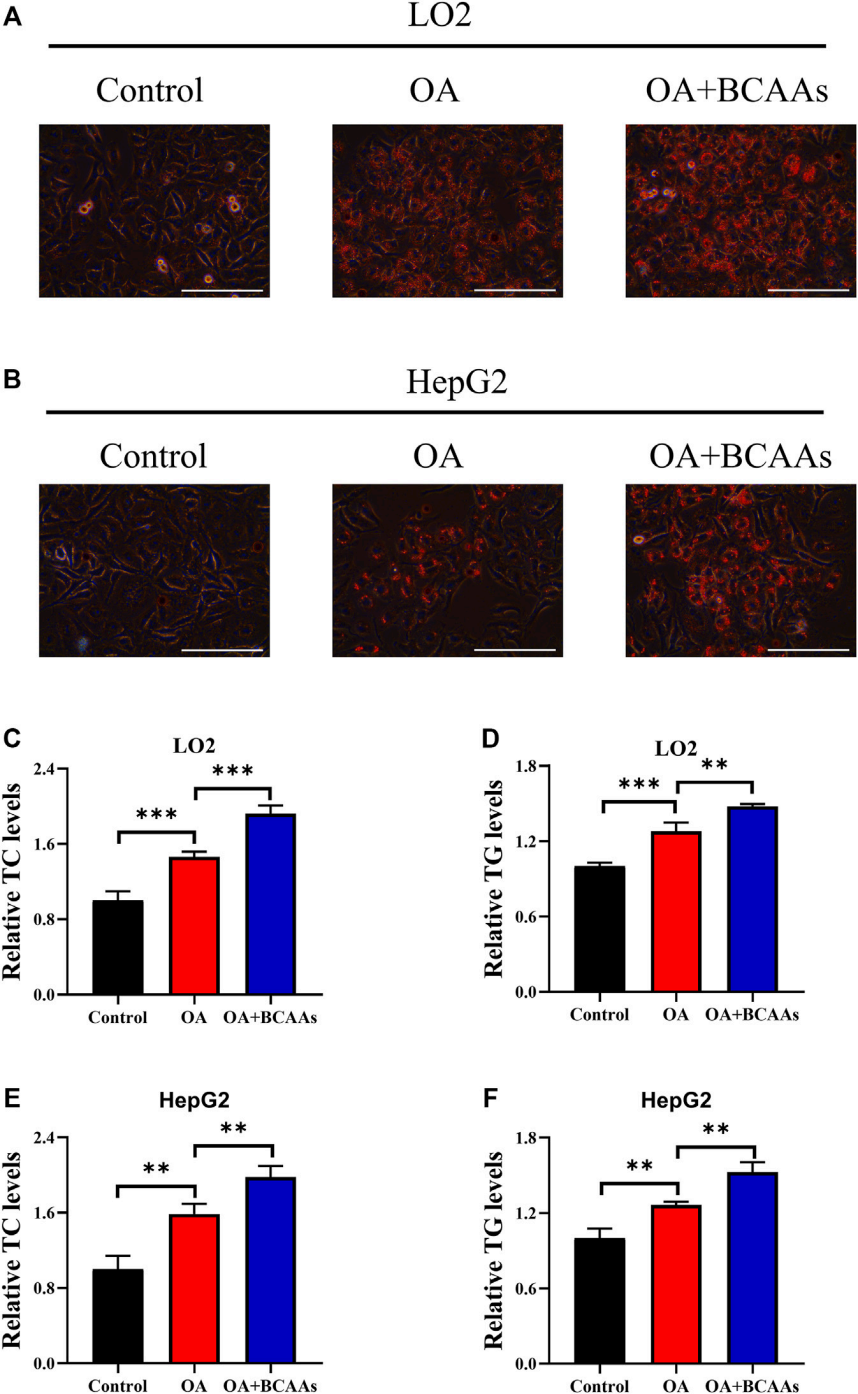
BCAA metabolism contributes to OA-induced hepatic steatosis. **(A–F)** LO2 and HepG2 cells were incubated with 0.5 mm Oleic acid with or without 10 mm BCAAs for 48 h. Intracellular lipid droplets were detected by Oil red O staining. Scale bar = 400 μM **(A,B)**. Intracellular TC and TG levels in LO2 **(C,D)** and HepG2 **(E,F)** cells were detected. Data are shown as the mean ± SD of three independent experiments. **p < 0.01, ***p < 0.001.

To explore the role of BCAA metabolism in OA-induced lipid accumulation, LO2 and HepG2 cells were incubated with oleic acid in the presence or absence of 10 mM BCAAs for 48 h. Formation of lipid droplets in cells was detected by Oil Red O staining. As shown in Figures 1A,B, oleic acid treatment induced lipid accumulation. Supplementation of BCAAs (10 mm valine, leucine and isoleucine) and oleic acid further facilitated lipid accumulation compared with those treated with oleic acid or control group. Triglycerides (TC) and cholesterol (TG) are two important constituents of lipid fraction (Neuman et al., 2014). Therefore, intracellular levels of TC and TG were examined. Our results showed that oleic acid treatment increased the content of TC and TG in LO2 and HepG2 cells, which could be further augmented by the supplementation of BCAAs (Figures 1C–F). These results indicated that BCAA metabolism could contribute to OA-induced lipid accumulation.

### BCATc inhibition alleviates OA-induced hepatic steatosis

**FIGURE 2:**
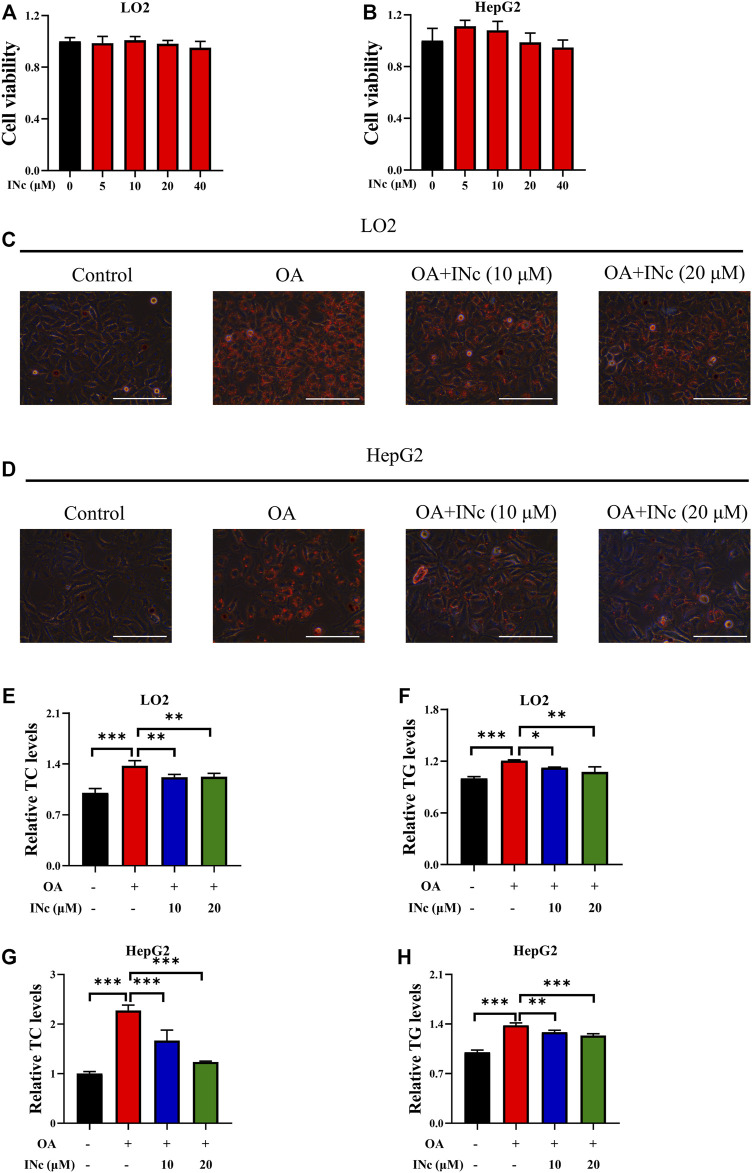
BCATc Inhibition alleviates OA-induced hepatic steatosis. **(A,B)** LO2 and HepG2 cells were incubated with indicated concentrations of BCATc Inhibitor 2 for 48 h and MTT assay was performed. **(C–H)** LO2 and HepG2 cells were incubated with 0.5 mm Oleic acid and 0, 10, 20 µM BCATc Inhibitor 2 for 48 h. Intracellular lipid droplets were detected by Oil red O staining. Scale bar = 400 μM **(C,D)**. Intracellular TC and TG levels in LO2 **(E, F)** and HepG2 **(G–H)** cells were detected. Data are shown as the mean ± SD of three independent experiments. *p < 0.05, **p < 0.01, ***p < 0.001. INc: BCATc Inhibitor 2.

BCATc Inhibitor 2 (INc), an inhibitor of BCATc, can be synthesized from coumarin derivative (Hu et al., 2006) and its chemical structure is shown in Supplementary Figure S1A. To assess the cytotoxicity of BCATc Inhibitor 2, LO2 and HepG2 cells were incubated with indicated concentrations of BCATc Inhibitor 2 for 48 h and MTT assay was performed. The results showed that BCATc Inhibitor 2 had no obvious cytotoxicity on LO2 and HepG2 cells at indicated concentrations (Figures 2A,B). To investigate the inhibitory effect of BCATc Inhibitor 2 on OA-induced lipid accumulation, cells were incubated with oleic acid in the presence or absence of BCATc Inhibitor 2. Oil Red O staining results showed that BCATc Inhibitor 2 alleviated OA-induced lipid accumulation (Figures 2C,D). Additionally, BCATc Inhibitor 2 treatment alleviated oleic acid-induced up-regulation of intracellular levels of TG and TC (Figures 2E–H). These results indicate that BCATc is involved in OA-induced lipid accumulation.

### Inhibiting BCATm has no effect on OA-induced hepatic steatosis

**FIGURE 3:**
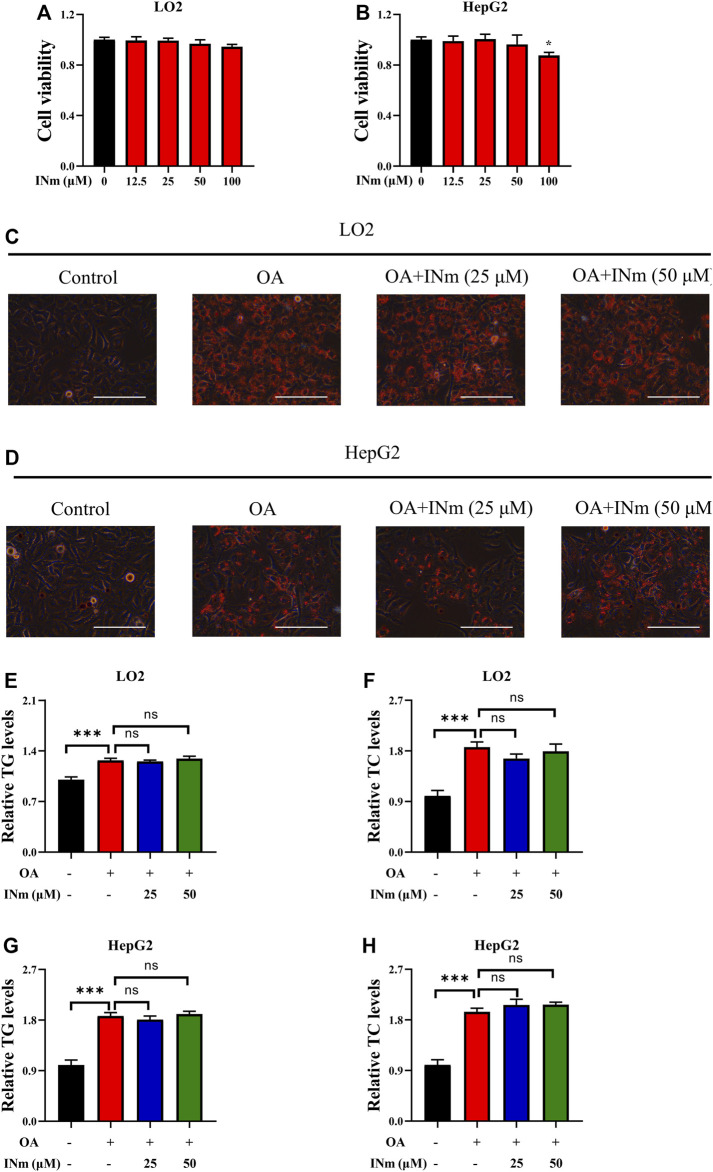
Inhibiting BCATm shows no effect on OA-induced hepatic steatosis. **(A,B)** LO2 and HepG2 cells were treated with indicated concentrations of BCAT-IN-2 for 48 h and MTT assay was performed. **(C–H)** LO2 and HepG2 cells were incubated with 0.5 mm Oleic acid and 0, 25, 50 µM BCAT-IN-2 for 48 h. Intracellular lipid droplets were detected by Oil red O staining. Scale bar = 400 μM **(C,D)**. Intracellular TC and TG levels in LO2 **(E,F)** and HepG2 **(G–H)** cells were detected. Data are shown as the mean ± SD of three independent experiments. ***p < 0.001, ns *p* > 0.05. INm: BCAT-IN-2.

BCAT-IN-2 (INm) is an inhibitor of mitochondrial branched-chain amino acid transferase (Bertrand et al., 2015) and its chemical structure is shown in Supplementary Figure S1B. To assess the cytotoxicity of BCAT-IN-2, LO2 and HepG2 cells were incubated with different concentrations of BCAT-IN-2 and MTT assay was performed. The results showed that BCAT-IN-2 had no obvious cytotoxicity on LO2 cells at indicated concentrations and HepG2 cell viability was slightly inhibited at the concentration of 100 µM (Figures 3A,B). To investigate the inhibitory effect of BCAT-IN-2 on OA-induced lipid accumulation, cells were incubated with oleic acid in the presence or absence of BCAT-IN-2. Oil Red O staining results showed that BCAT-IN-2 had no effect on OA-induced lipid accumulation (Figures 3C,D). Furthermore, OA-induced TG and TC production was not changed following BCAT-IN-2 treatment (Figures 3E–H). These results indicate that BCATm is not involved in OA-induced lipid accumulation.

### BCATc inhibitor 2 alleviates OA-induced expression of lipogenesis-related genes

**FIGURE 4:**
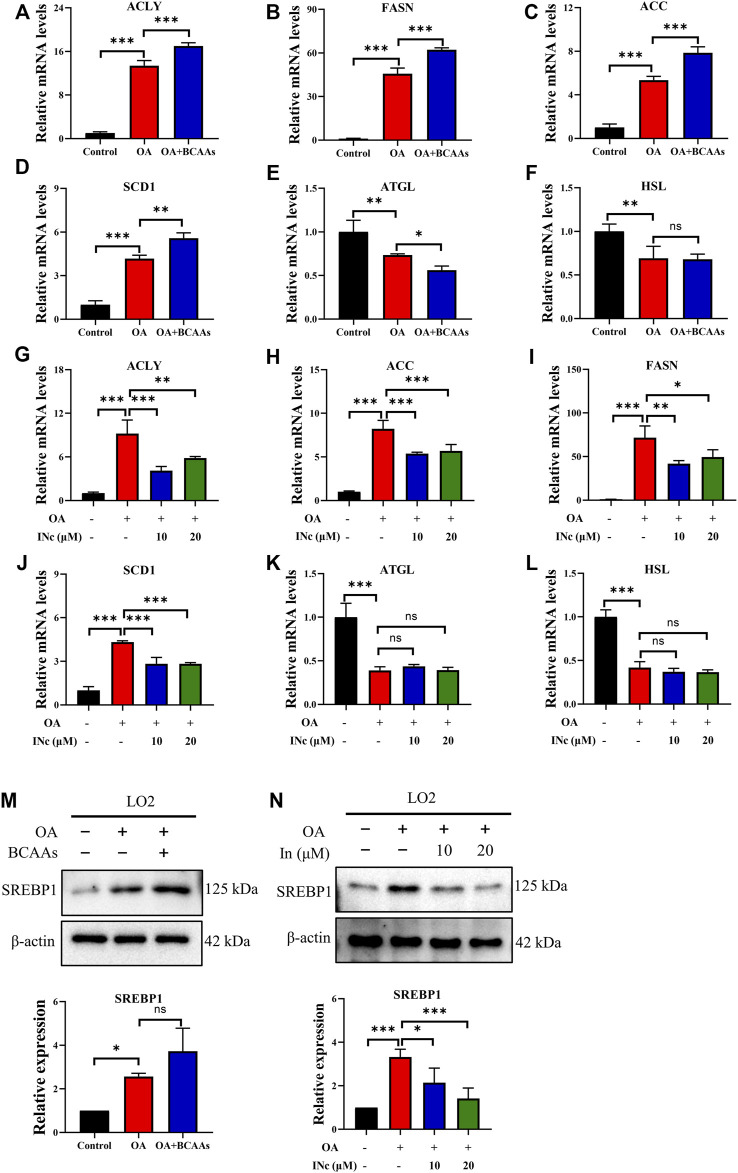
BCATc Inhibitor 2 alleviates OA-induced expression of lipogenesis-related genes. **(A–F)** LO2 cells were incubated with 0.5 mm Oleic acid with or without 10 mm BCAAs for 48 h qPCR was performed to determine the expression of ACLY **(A)**, FASN **(B)**, ACC **(C)**, SCD1 **(D)**, ATGL **(E)**, HSL **(F)**. **(G–M)** LO2 cells were incubated with 0.5 mM Oleic acid and 0, 10, 20 µM BCATc Inhibitor 2 for 48 h qPCR was performed to determine the expression of ACLY **(G)**, FASN **(H)**, ACC **(I)**, SCD1 **(J)**, ATGL **(K)**, HSL **(L)**. **(M,N)** LO2 cells were incubated with 0.5 mm Oleic acid with or without 10 mm BCAAs or BCATc Inhibitor 2 for 48 h. The expression of SREBP1 was detected by western blot. Data are shown as the mean ± SD of three independent experiments. *p < 0.05, **p < 0.01, ***p < 0.001, ns *p* > 0.05. INc: BCATc Inhibitor 2.

To further investigate the molecular mechanism underlying the inhibitory effect of BCATc Inhibitor 2 on OA-induced lipid accumulation, expression of lipogenesis and lipolysis-related genes was detected. qPCR analysis showed that mRNA levels of lipogenesis-related genes such as ACLY, ACC, FASN and SCD1 were up-regulated following oleic acid treatment. Supplementation of BCAAs further increased the expression of ACLY, FASN and SCD1 (Figures 4A–D). On the contrary, mRNA levels of lipolysis-related gens such as ATGL and HSL were down-regulated following oleic acid treatment. However, supplementation of BCAAs had no effect on the mRNA level of these genes (Figures 4E,F). Moreover, treatment of BCATc Inhibitor 2 decreased the expression of lipogenesis-related genes induced by oleic acid (Figures 4G–J). The expression of ATGL and HSL was not affected by BCATc Inhibitor 2 (Figures 4K,L). SREBP1 is a key transcription factor regulating the synthesis of lipid and cholesterol. Therefore, the expression of SREBP1 was detected by western blot. The results showed that treatment with oleic acid or the combination of oleic acid and BCAAs up-regulated the expression of SREBP1 (Figures 4M). On the contrary, BCATc Inhibitor 2 treatment down-regulated the expression of SREBP1 induced by oleic acid (Figures 4N). These results suggest that BCATc Inhibitor 2 inhibited OA-induced lipid accumulation by suppressing lipogenesis.

### BCATc inhibitor 2 alleviates OA-induced cell apoptosis

**FIGURE 5:**
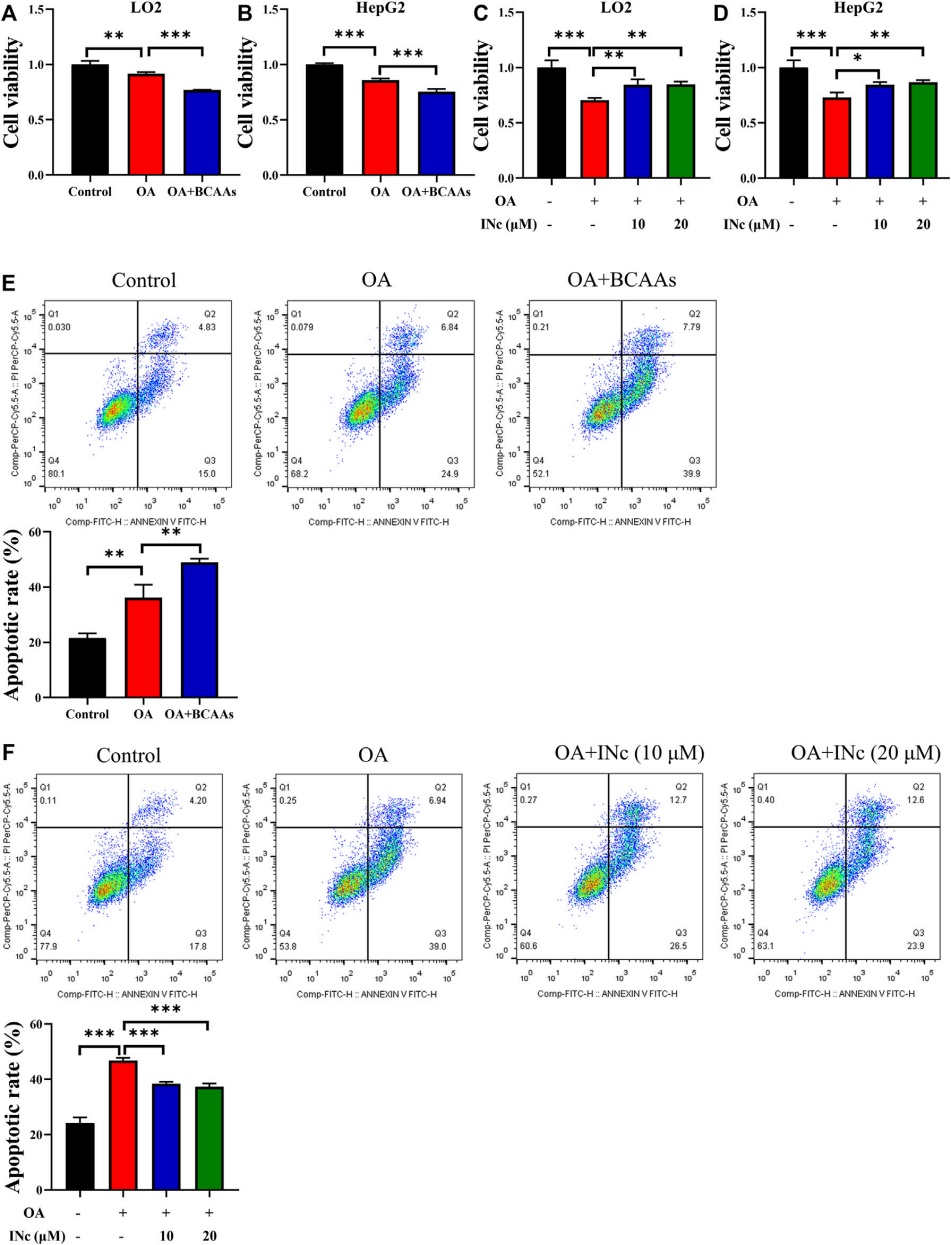
BCATc Inhibitor 2 alleviates OA-induced cell apoptosis. **(A,B)** LO2 and HepG2 cells were incubated with 0.5 mm Oleic acid with or without 10 mm BCAAs for 48 h. Cell viability was detected by MTT assay. **(C,D)** LO2 and HepG2 cells were incubated with 0.5 mm Oleic acid in the presence 0, 10, 20 µM BCAT-IN-2 for 48 h. Cell viability was detected by MTT assay. **(E)** LO2 cells were incubated with 0.5 mm Oleic acid with or without 10 mm BCAAs for 48 h. Apoptotic cell rate was determined by Annexin V/PI staining and cytometry based on Q2+Q3. **(F)** LO2 cells were incubated with 0.5 mm Oleic acid and 0, 10, 20 µM BCATc Inhibitor 2 for 48 h. Apoptotic cell rate was determined by Annexin V/PI staining and cytometry based on Q2+Q3. Data are shown as the mean ± SD of three independent experiments. **p < 0.01, ***p < 0.001. INc: BCATc Inhibitor 2.

Apoptosis is a key feature in the pathogenesis of oleic acid-induced NAFLD (Xie et al., 2016). Therefore, we detected whether BCATc Inhibitor 2 could protect cell against oleic acid toxicity. MTT assay results showed that supplementation of BCAAs in the presence of oleic acid further inhibited cell viability compared with those treated with oleic acid or control group (Figures 5A,B). On the contrary, BCATc Inhibitor 2 treatment alleviated the inhibitory effect of oleic acid on cell viability (Figures 5C,D). Further, cell apoptosis was examined by Annexin V/PI staining. Cell apoptotic rate in OA group was approximately 36%. Supplementation of BCAAs in the presence of oleic acid resulted in higher cell apoptotic rates compared with those treated with oleic acid or control group (Figures 5E,F). On the contrary, BCATc Inhibitor 2 treatment ameliorated cell apoptosis induced by oleic acid (Figures 5G,H), indicating that BCATc Inhibitor 2 could protect hepatic cells against oleic acid cytotoxicity.

### BCATc inhibitor 2 alleviates OA-induced mitochondrial membrane potential disruption and mitochondrial ROS production

**FIGURE 6:**
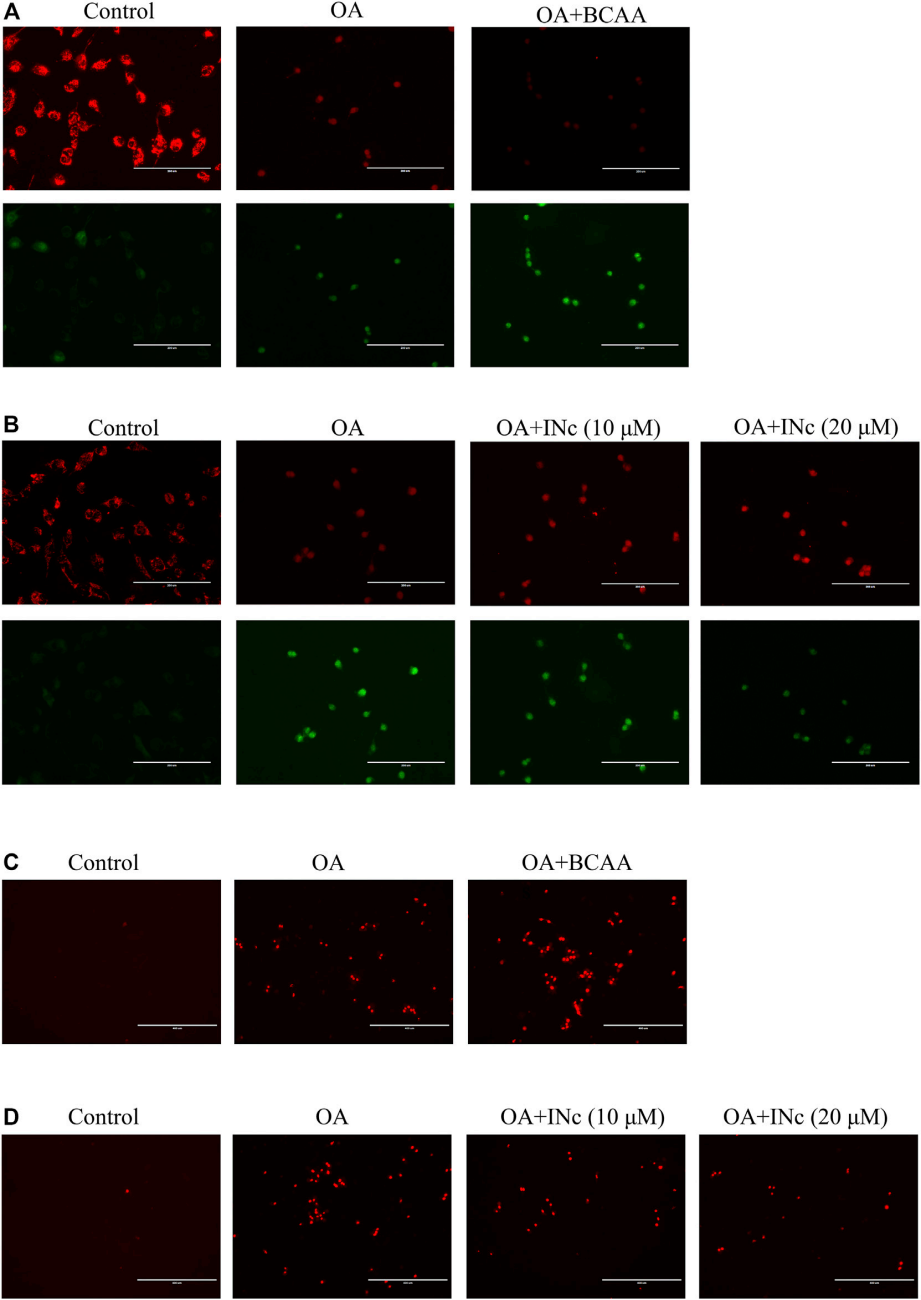
BCATc inhibitor 2 alleviates OA-induced mitochondrial membrane potential disruption and intracellular ROS production. **(A,B)** LO2 cells were incubated with 0.5 mm Oleic acid with or without 10 mm BCAAs or BCATc Inhibitor 2 for 48 h. Mitochondrial membrane potential was determined by JC-1 staining. Scale bar = 200 µM. **(C-D)** LO2 cells were incubated with 0.5 mm Oleic acid with or without 10 mm BCAAs or BCATc Inhibitor 2 for 48 h. Mitochondrial ROS level was detected by MitoSOX staining. INc: BCATc Inhibitor 2.

It has been reported that mitochondrial dysfunction is a hallmark of NAFLD (Nassir and Ibdah, 2014). Here, JC-1 staining was performed to detect mitochondrial membrane potential. The ratio of red/green fluorescence indicate the change in mitochondrial membrane potential. As shown in Figure 6, compared with control group, oleic acid treatment disrupted mitochondrial membrane potential in LO2 cells. Co-treatment with BCAAs further exacerbated mitochondrial membrane potential disruption. However, co-treatment with BCATc Inhibitor 2 prevented OA-induced disruption of mitochondrial membrane potential.

Mitochondrial membrane potential disruption was reported to facilitate ROS production (Suski et al., 2018). Next, mitochondrial ROS level was determined with MitoSOX staining. As shown in Figures 6C,D, OA treatment increased mitochondrial ROS level. Co-treatment with BCAAs further promoted the production of mitochondrial ROS. However, co-treatment with BCATc Inhibitor 2 alleviated the OA-induced mitochondrial ROS production in LO2 cells. All these results indicate that BCATc Inhibitor 2 ameliorated oleic acid-induced mitochondrial dysfunction in hepatic cells.

### BCATc inhibitor 2 antagonizes OA-induced activation of Bcl2/Bax/Caspase axis and AKT/JNK signaling

**FIGURE 7:**
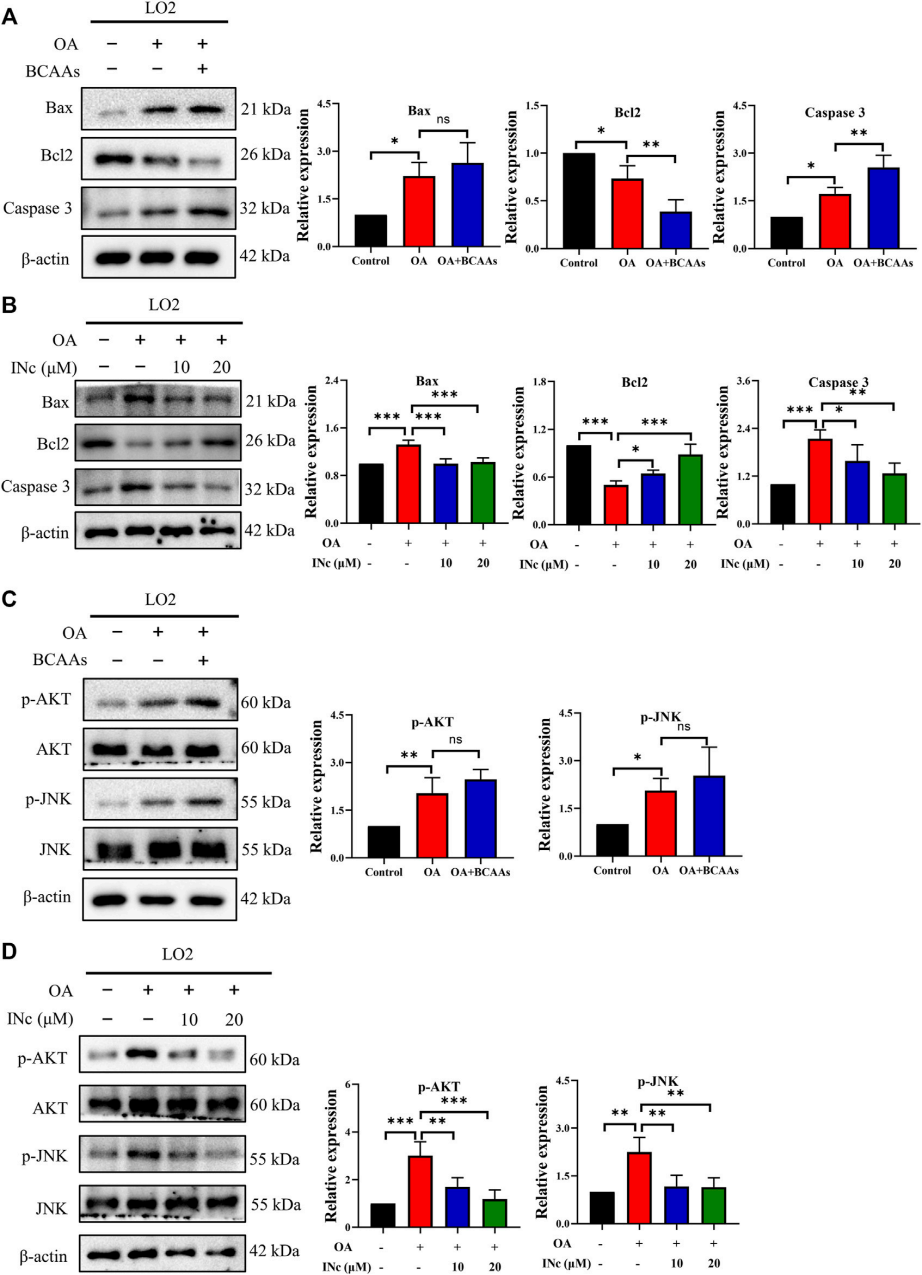
BCATc Inhibitor 2 antagonizes OA-induced activation of Bcl2/Bax/Caspase axis and AKT/JNK signaling. **(A–D)** LO2 cells were incubated with 0.5 mm Oleic acid with or without 10 mm BCAAs or BCATc Inhibitor 2 for 48 h. Western blot was performed to detect the expression of indicated proteins. Data are shown as the mean ± SD of three independent experiments. *p < 0.05, **p < 0.01, ***p < 0.001, ns *p* > 0.05. INc: BCATc Inhibitor 2.

To further investigate the mechanism regarding the protective effect of BCATc Inhibitor 2 against OA-induced apoptosis, the expression of apoptosis-related protein were assessed by western blot. As shown in Figures 7A,B and Supplementary Figures S2A,S2B, OA treatment increased the expression of Bax, Caspase 3 and Caspase 9 and decreased the expression of Bcl2. Supplementation of BCAAs further up-regulated the expression of Caspase 3 and inhibited the expression of Bcl2. On the contrary, co-treatment with BCATc inhibitor 2 prevented OA-induced upregulation of Bax, Caspase 3 and Caspase 9 as well as downregulation of Bcl2. These results indicate that BCATc Inhibitor 2 alleviates OA-induced apoptosis *via* Bcl2/Bax/Caspase axis.

Previous studies showed that JNK and AKT pathway are important regulators of apoptosis (Dhanasekaran and Reddy, 2017; Meng et al., 2017). As shown in Figures 7C,D, expression of p-AKT and p-JNK was up-regulated following OA treatment. Supplementation of BCAAs in the presence of OA further promoted the phosphorylation of JNK and AKT. However, co-treatment with BCATc inhibitor 2 alleviated OA-induced phosphorylation of AKT and JNK. These results suggest that BCATc Inhibitor 2 alleviates OA-induced apoptosis by inhibiting AKT and JNK signaling pathway.

## Discussion

In this study, we investigate the role of BCAAs metabolism in the progression of NAFLD and the protective effect of BCATc Inhibitor 2 against NAFLD with its underlying mechanism. Oleic acid is a mono-unsaturated omega-9 fatty acid that is used to establish *in vitro* NAFLD model (Xie et al., 2016). Consistent with previous findings, LO2 and HepG2 cells treated with oleic acid showed formation of lipid droplets and higher apoptotic rate, suggesting that *in vitro* NAFLD model was successfully established.

Previous studies have showed strong correlation between BCAAs and lipid metabolism (Newgard, 2012). It was found that BCAAs deprivation inhibited lipogenesis in C2C12 myotubes (Karvinen et al., 2022). *in vivo* experiments showed that BCAAs aggravated obesity-related lipid metabolic disorders in mice and regulated fat disposition in pig (Heng et al., 2020; Zhao et al., 2020). Here, we showed that supplementation of BCAAs promoted lipid accumulation in oleic acid-induced LO2 and HepG2 cells. Intracellular level of TG and TC also increased following supplementation of BCAAs. Cytosolic BCATc and mitochondrial BCATm are two isoforms of branched chain amino acid transaminase that share similar enzymatic functions. However, recent studies indicated that BCATc and BCATm had different regulatory mechanisms and physiological functions (Toyokawa et al., 2021). BCATc Inhibitor 2 can be synthesized from coumarin derivative, a natural compound that exists in vascular plants (Cooke et al., 1997). Intriguingly, inhibiting BCATc by BCATc Inhibitor 2 significantly alleviated Oleic acid-induced formation of lipid droplets and up-regulation of TC and TG. In contrast, BCATm inhibition by BCAT-IN-2 showed no effect on lipid accumulation. These results indicate that BCATc participates in Oleic acid-induced NAFLD model in hepatic cells.

Lipid homeostasis is maintained by the balance between lipogenesis and lipolysis (Lelliott and Vidal-Puig, 2004). Lipogenesis is mainly regulated by ACLY, FASN, SCD and ACC, while ATGL and HSL participates in lipolysis (Miyoshi et al., 2008). It was found that supplementation of BCAAs increased the expression of lipogenesis-related genes and BCATc Inhibitor 2 treatment decreased the expression of lipogenesis-related genes in LO2 and HepG2 cells. Though the expression of lipolysis-related genes were not affected either by supplementation of BCAAs or BCATc Inhibitor 2 treatment. SREBP1 is a key transcriptional factor that regulates the expression of lipogenesis-related genes (Ruiz et al., 2014). Supplementation of BCAAs up-regulated the expression of SREBP1 while BCATc Inhibitor 2 treatment down-regulated the expression of SREBP1. All these results suggest that enhanced BCAA metabolism increased lipid accumulation by promoting lipogenesis.

Lipotoxicity-induced apoptosis in hepatic is a hallmark of NAFLD (Kusminski et al., 2009). Supplementation of BCAAs further inhibited cell viability and induced cell apoptosis in oleic acid-induced NAFLD model. On the contrary, BCATc Inhibitor 2 treatment alleviated oleic acid-induced cell viability inhibition and apoptosis in LO2 and HepG2 cells. Bcl2 and Bax are anti-apoptotic and apoptotic proteins (Pawlowski and Kraft, 2000), and Caspase 3 and Caspase 9 are the main effector of apoptosis (Yu et al., 2014). Oleic acid treatment and supplementation of BCAAs increased the expression of Bax and Caspase 3 and decreased the expression of Bcl2. On the contrary, BCATc Inhibitor 2 treatment alleviated oleic acid-induced up-regulation of Bax, Caspase 3 and Caspase 9 as well as down-regulation of Bcl2. These results suggest that BCATc inhibition attenuated oleic acid-induced apoptosis through Bcl2/Bax/Caspase axis. JNK is a member of mitogen-activated protein kinase family that participates in cell growth, survival and death. AKT/JNK signaling was reported to induce apoptosis under stressed condition in Bcl2/Bax-dependent manner (Lei and Davis, 2003; Li et al., 2018). Here, it was found that oleic acid up-regulated the expression of p-AKT and p-JNK in LO2 cells. Supplementation of BCAAs further increased the expression of p-AKT and p-JNK. In contrast, BCATc Inhibitor 2 treatment prevented oleic acid-induced up-regulation of p-AKT and p-JNK, indicating BCATc Inhibitor 2 alleviated oleic acid-induced apoptosis by inhibiting AKT/JNK signaling.

Mitochondrial dysfunction is closed related with the progression of NAFLD, which promotes ROS generation, lipid peroxidative and eventually cell death (Begriche et al., 2006). We found that OA treatment resulted in mitochondrial membrane potential disruption and mitochondrial ROS production, indicating that mitochondrial function was impaired. Supplementation of BCAAs further disrupted mitochondrial function, as indicated with lowered mitochondrial membrane potential disruption and increased intracellular ROS level. However, BCATc Inhibitor 2 treatment alleviated OA-induced mitochondrial dysfunction. These results suggest that BCATc Inhibitor 2 ameliorates OA-induced cell death by preserving mitochondrial function.

In conclusion, we demonstrate that inhibiting BCATc by BCATc Inhibitor 2 alleviates Oleic acid-induced lipid accumulation and apoptosis in hepatic cells. Molecularly, BCATc Inhibitor 2 down-regulated lipogenesis-related genes and inhibited AKT/ERK signaling as well as Bcl2/Bax/Caspase axis. Our results shed new light on the role of BCAA metabolism in NAFLD and propose that BCATc Inhibitor 2 could be a candidate drug for the treatment of NAFLD in clinic.

## Data Availability

The original contributions presented in the study are publicly available. This data can be found here: https://www.jianguoyun.com/p/DSKCqj8Q8ajRChigguIEIAA.

## Abbreviations

BCAA, branched chain amino acid; BCAT, branched-chain amino acid transferase; INc, BCATc inhibitor 2; INm; BCAT-IN-2; NAFLD, nonalcoholic fatty liver disease; NASH, nonalcoholic steatohepatitis; OA, oleic acid; TG, cholesterol; TC, triglycerides.
