# Supplementary material for: BCATc inhibitor 2 ameliorated mitochondrial dysfunction and apoptosis in oleic acid-induced non-alcoholic fatty liver disease model
Source: Front Pharmacol. 2022 Oct 28;13:1025551. doi: 10.3389/fphar.2022.1025551 (PMC9650408; doi:10.3389/fphar.2022.1025551)
Supplement: Supplementary file 1 [file DataSheet1.PDF]

Supplementary Figure 1

A

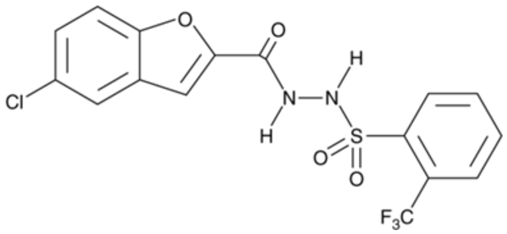

B

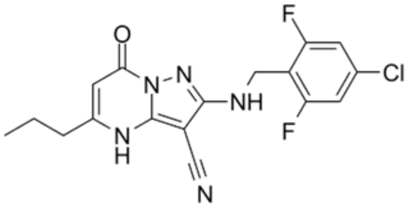

Supplementary Figure 1. Chemical structure of BCATc Inhibitor 2 **(A)** and BCAT-IN-2 **(B)**.

Supplementary Figure 2

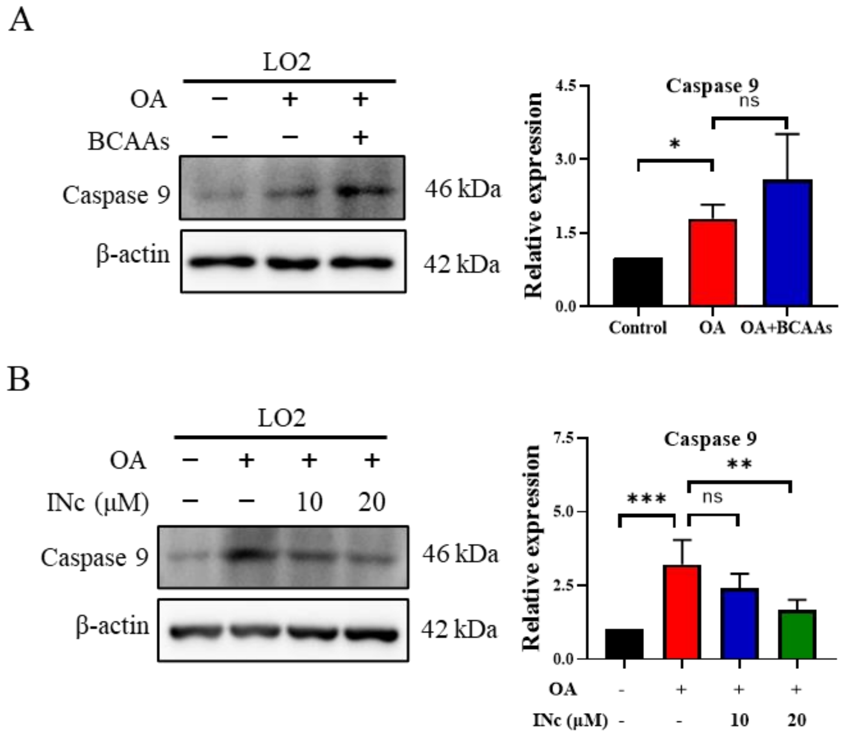

Supplementary Figure 2. **(A)** LO2 cells were incubated with 0.5 mM Oleic acid with or without 10 mM BCAAs for 48 h. **(B)** LO2 cells were incubated with 0.5 mM Oleic acid with or without BCATc Inhibitor 2 for 48 h. Western blot was performed to detect the expression of Caspase 9. Data are shown as the mean ± SD of three independent experiments. *p<0.05, **p<0.01, ***p<0.001, ns p>0. INc: BCATc Inhibitor 2.
